# Barriers-on-chips: Measurement of barrier function of tissues in organs-on-chips

**DOI:** 10.1063/1.5023041

**Published:** 2018-06-26

**Authors:** Yusuf B. Arık, Marinke W. van der Helm, Mathieu Odijk, Loes I. Segerink, Robert Passier, Albert van den Berg, Andries D. van der Meer

**Affiliations:** 1 Department of Applied Stem Cell Technologies, University of Twente, 7522 NB Enschede, The Netherlands; 2 BIOS Lab on a Chip Group, MESA+ Institute for Nanotechnology, Max Planck Center for Complex Fluid Dynamics, University of Twente, 7522 NB Enschede, The Netherlands; 3 Department of Anatomy and Embryology, Leiden University Medical Center, 2333 ZA Leiden, The Netherlands

## Abstract

Disruption of tissue barriers formed by cells is an integral part of the pathophysiology of many diseases. Therefore, a thorough understanding of tissue barrier function is essential when studying the causes and mechanisms of disease as well as when developing novel treatments. *In vitro* methods play an integral role in understanding tissue barrier function, and several techniques have been developed in order to evaluate barrier integrity of cultured cell layers, from microscopy imaging of cell-cell adhesion proteins to measuring ionic currents, to flux of water or transport of molecules across cellular barriers. Unfortunately, many of the current *in vitro* methods suffer from not fully recapitulating the microenvironment of tissues and organs. Recently, organ-on-chip devices have emerged to overcome this challenge. Organs-on-chips are microfluidic cell culture devices with continuously perfused microchannels inhabited by living cells. Freedom of changing the design of device architecture offers the opportunity of recapitulating the *in vivo* physiological environment while measuring barrier function. Assessment of barriers in organs-on-chips can be challenging as they may require dedicated setups and have smaller volumes that are more sensitive to environmental conditions. But they do provide the option of continuous, non-invasive sensing of barrier quality, which enables better investigation of important aspects of pathophysiology, biological processes, and development of therapies that target barrier tissues. Here, we discuss several techniques to assess barrier function of tissues in organs-on-chips, highlighting advantages and technical challenges.

## INTRODUCTION

The human body contains numerous barriers, some of which separate the internal environment from the external environment and others that separate different compartments inside the body. These barriers are found in, for example, skin, airways, brain, eye, and blood vessels, and they maintain homeostasis by regulating the interactions between the compartments that they separate. Moreover, barriers such as the blood-brain barrier (BBB), blood retinal barrier (BRB), and the pulmonary air-liquid interface (ALI) are highly selective to prevent toxins from affecting vital organs. Disruption and dysfunction of such tissues are of major importance in the pathophysiology of many human diseases (e.g., BBB disruption in multiple sclerosis, meningitis, encephalitis,^1^ BRB disruption in diabetic retinopathy, macular degeneration,^2^ ALI disruption in pulmonary edema^3^).

It is well known that the biophysical and biochemical tissue microenvironment in terms of blood flow, interstitial flow, tissue shape and curvature, mechanical strain, paracrine signaling, and the local interaction between various cell types all play important roles in maintaining or altering the barrier function of tissues.^4–7^ Current *in vitro* methods fail to provide this dynamic physicochemical microenvironment. Therefore, there is a strong need for advanced *in vitro* systems that allow the controlled and routine inclusion of a realistic tissue microenvironment when studying the barrier function of cultured cells.

Organs-on-chips are a new class of microphysiological *in vitro* models of human organs and tissues that rely on culturing cells in a well-controlled microenvironment that has been engineered to include key physical and biochemical parameters.^5,8–14^ Organs-on-chips show great promise in mimicking human tissues and organs and are being used in both fundamental and translational biomedical research. For organs-on-chips to be valuable as research tools, it is essential that the state of the cells in an organ-on-a-chip can be probed and quantified in various ways. Some of the most often measured physiological parameters in the current generation of organs-on-chips are related to tissue barrier function. Importantly, measuring permeability in organs-on-chips is associated with unique challenges that are related to their small size, low volumes, and dynamic nature. It is essential to understand these challenges and to analytically characterize the organ-on-a-chip system that is being used.

In this review, we give examples of organ-on-chip systems in which various parameters related to barrier function were routinely measured. We discuss the advantages and challenges of measuring barrier function in organ-on-a-chip systems, and we give practical pointers for avoiding the most common measurement errors. Although active receptor-mediated transport is very important in physiology and drug discovery, and organ-on-chip systems show great promise in realistically mimicking physiological expression profiles of receptors,^15–17^ active transportation of molecules will not be discussed in this review. The assessment of cellular active transport *in vitro* has been discussed elsewhere,^18^ and the same is true for the potential role of organs-on-chips in drug discovery.^19^

## CELL CULTURING PLATFORMS FOR BARRIER ASSESSMENT

Prior to giving examples and information about the methods to quantify barrier integrity in organs-on-chips, the section on “Conventional cell culturing systems” gives an overview of methods in conventional *in vitro* models which commonly use Transwell systems. Since fundamental principles of these techniques are similar in different platforms, basics discussed below will help to understand the techniques in organ-on-a-chip platforms.

### Conventional cell culturing systems

**FIG. 1.**
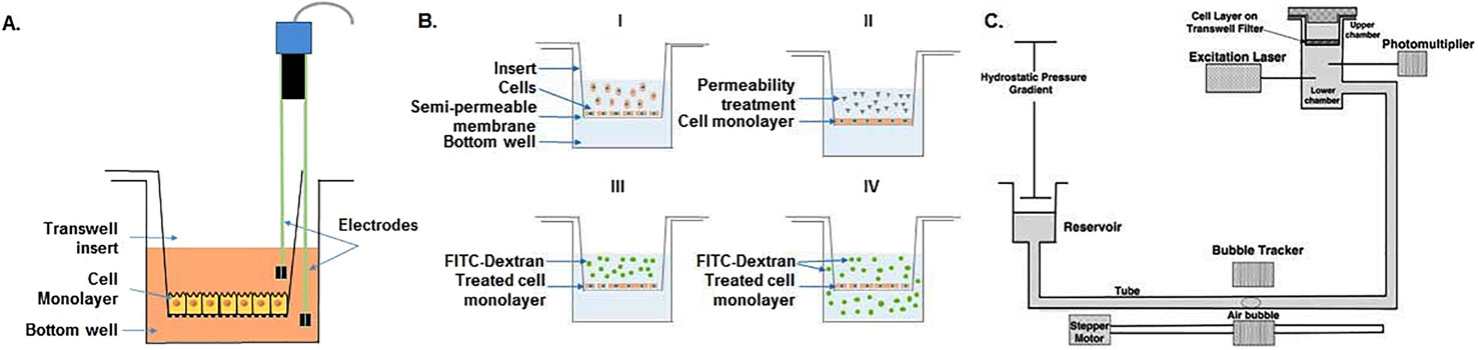
**Conventional barrier assessment methods.** (A) TEER measurements in Transwell systems uses two electrode pairs that are submerged in different compartments, measuring resistance of the cellular monolayer seeded onto the membrane. (B) Evaluating barrier by means of transport of fluorescently labeled dextran starts with cells cultured to a monolayer (B-I), then treated with a disease stimulus to change their permeability (B-II). After that FITC-dextran is added to the insert (B-III), and samples can be collected from the bottom compartment to measure the integrity of the cell layer (B-IV). Schematics of experimental setup for measuring hydraulic conductivity (C) starts with Transwell insert sealed in a chamber. For water transport, the reservoir is lowered to create a pressure gradient across the cell layer, then the water flux across the cell monolayer is measured using bubble tracker, and the hydraulic conductivity is determined accordingly [Reproduced with permission from Li *et al.*, Ann. Biomed. Eng. **38**, 2499 (2010). Copyright 2010 Springer Nature.]

Because barriers are so important in health and disease, experimental *in vitro* tools that can be used to quantify and characterize the barrier function of cells and tissues are currently widely used. Most conventional techniques typically make use of a Transwell cell culture system, which relies on a tissue-culture plate with two culture compartments—the well and the insert—that are separated by a synthetic porous membrane (Fig. 1). When cells are grown on the synthetic membrane, their barrier function can be assessed by measuring various parameters. In addition to assessing barriers by imaging cell-cell junction proteins using fluorescent and electron microscopy, there are various parameters that can be measured to evaluate the barrier function of cultured cell layers: electrical resistance, mass transport, and hydraulic conductivity; all three parameters will be discussed briefly below.

Transepithelial/endothelial electrical resistance (TEER) is one of the widely used methods for evaluating barriers; it gives an indication of the tightness of cell-cell junctions in the paracellular space by means of electrical resistance across a monolayer. For measurements, a commercially available Epithelial Voltohmmeter (EVOM) is often used which consists of a pair of legs with two pairs of electrodes. One of these legs is placed in the upper compartment whereas the other is submerged into the culture medium in the lower compartment [Fig. 1(A)]. Each of these legs contains an electrode to apply a current to the barrier, while the other electrode of the pair is used to measure the resulting voltage over the barrier. Since direct current can be damaging to the cells and electrodes, an alternating current (AC) of 10 *μ*A amplitude with a square waveform and a relatively low frequency (typically 12.5 Hz) is applied. For analyzing TEER, first the resistance of the permeable membrane only (without cells, R_membrane_) is measured, followed by a measurement of the resistance across the cell layer on the membrane (R_total_). The specific resistance of the cell layer (R_cells_) is then calculated by subtracting the blank resistance from the total resistance [Eq. (1)] and normalizing for cell culture area (A_membrane_) [Eq. (2)]^20^

$$R_{\text{cells}}=R_{\text{total}}-R_{\text{membrane}},$$ (1)

$${\text{TEER}}_{\text{cells}}=\;R_{\text{cells}}\times A_{\text{membrane}}.$$ (2)

Readings of TEER are highly dependent on the electrode positions, and careful handling while placing the electrodes is important as it might disturb the cell monolayer. In addition, having a uniform current density generated by the electrodes has an impact on TEER values. To accurately provide that, correct type of electrode systems should be chosen. For instance, classical STX2/Chopstick electrode cannot be used for a relatively large membrane (i.e., 24 mm diameter) in tissue culture inserts.^21^ This may result in overestimation of the TEER value, and as an alternative in this case, a better suited EndOhm chambers can be used to cover larger areas.^22^ TEER is a sensitive, non-invasive method and with dedicated measurement systems, it is possible to monitor live cells during various stages of growth, differentiation, or experimental treatment.

Barrier integrity of cells can also be assessed by measuring the paracellular diffusive transport of tracer compounds of various molecular weights. While TEER measures the ion flux through the barrier, studies of paracellular transport can give more detailed information about the paracellular spacing when using different tracers of defined molecular weights. Tracers are typically added to the insert, and their diffusion over the cell layer into the well is tracked over time to determine the molecular flux [Fig. 1(B)]. Tracers can be radioactively, fluorescently, or enzymatically labeled. Radiolabeling is capable of detecting subtle changes in a barrier; however, they require special handling and safety measures, and their short half-life means that they cannot be stored for long periods. Therefore, this type of labeling is not usually preferred for barrier assessment.^23^ On the other hand, usage of enzymatic markers (e.g., horseradish peroxidase) has been reported for macromolecular diffusion. Low amounts of enzymes can still be sensitively quantified with the addition of sufficient amount of substrate and spectroscopically measuring the product of the catalyzed reaction, but the activity of enzymes can be affected by factors such as pH, temperature, and serum constituents thereby limiting its application.^24^ Due to ease of handling, non-radioactive, fluorescently labeled marker polysaccharides [e.g., fluorescein isothiocyanate (FITC)-labeled dextran] or proteins are widely used for permeability assays.^25^ Depending on the biological application, the size of tracer compounds can vary widely [e.g., inulin (5 kDa), mannitol (182 Da), albumin (67 kDa)].^20^ Despite ease-of-use, fluorescent tracers sometimes lack the required sensitivity to detect subtle changes in barriers due to poor specific activity (fluorescence/mg protein) or fluorophore instability.^24^ In general, it should be noted that the use of any tracer compounds may interfere with the transport process under study and may affect the barrier integrity as well as rendering the tested cells unusable for further experiments.^20^

Quantification of paracellular diffusive transport of tracer molecules typically starts with cell seeding to a Transwell membrane [Fig. 1(B-I)] followed by treatment of the cellular monolayer with a molecule of interest which would induce a change in permeability [Fig. 1(B-II)]. After treatment, a known concentration of a labeled tracer, such as FITC-dextran, is added to the insert [Fig. 1(B-III)] and over time, its diffusion over the cell layer is measured by taking repeated samples from the bottom well [Fig. 1(B-IV)]. The concentration of labeled dextran in individual samples can be calculated by measuring fluorescence intensity with a plate reader and standardizing against a calibration curve. If the increase in concentration is linear over time (which is typically only true in the initial stage of the experiment, when the concentration gradient between insert and well is still constant), the permeability coefficient of a solute can be calculated using the following equation:

$$P=\frac{1}{C_{i}}{\left(\frac{{dC}_{w}}{dt}\right)}_{0}\frac{V_{w}}{A},$$ (3)

where the permeability coefficient P is a function of C_i_, the initial concentration in the insert; (dC_w_/dt)_0_, the linear fit for the rate of increase in concentration at the start of the experiment; V_w_, the volume of the well; and A, the culture area.

In order to isolate the permeability coefficient of the cell layer (P_cell_) [Eq. (4)], a blank measurement in which permeability of the membrane (P_0_) was established according to the method above should be subtracted from the measured permeability of cells grown on the membrane (P_total_)

$$\frac{1}{P_{\text{cell}}}\;=\;\frac{1}{P_{\text{total}}}-\;\frac{1}{P_{0}}.$$ (4)

In addition to aforementioned techniques, measuring the flux of water across a cellular monolayer, also known as the hydraulic conductivity of a tissue, is another method to assess the barrier function of cultured cells. When performing measurements of hydraulic conductivity, water flux is facilitated by a defined pressure gradient. In addition, hydraulic conductivity can also be used to determine the optimal transmural pressure required to prevent delamination of endothelial cells from scaffold walls, which is a common challenge in micro-vessel engineering.^26–29^ Hydraulic conductivity can vary similar to permeability across cellular monolayers found in different locations in the human body (e.g., endothelial cells). *In vitro* endothelial monolayers may be optimized to produce tighter or leakier vessels to water by using different cell material and exposing the cells to different shear stresses and pressures to model different tissues, such as the tight blood-brain barrier or the permeable kidney glomerulus.^29–32^ *In vitro* measurements of hydraulic conductivity were demonstrated by Li *et al.* using a Transwell system [Fig. 1(C)].^33^ First, cells were grown on the Transwell insert to a monolayer. The filter was then sealed within a chamber. This chamber was connected to a water reservoir by a Tygon and borosilicate glass tube [Fig. 1(C)]. A difference in hydrostatic pressure was created across the filters by adjusting the height difference between the reservoir and the fluid covering the cell layers. Flow of water across the cell layers was then measured by tracking the position of a bubble pre-inserted into the glass tube. Using the volumetric flow rate derived from the displacement of the air bubble, hydraulic conductivity (L_p_) can be calculated by [Eq. (5)]

$$L_{p}=\frac{J_{v}}{A\;\times \;\Delta p},$$ (5)

where J_v_ is the volumetric flow rate, A is the surface area of the Transwell insert, and Δp is the hydraulic pressure difference across the cell layer.

### Organ-on-a-chip systems

When using conventional *in vitro* systems, the barrier function of tissues is often found to be decreased compared to the physiological *in vivo* situation. For example, *in vivo* values of barrier tightness of the blood-brain barrier have been reported to be larger than what can be achieved with simple *in vitro* systems.^34^ Since inclusion of biochemical and mechanical stimuli that the cells would normally experience in their *in vivo* microenvironment has such an impact on their barrier function, there is a need for advanced conventional models that incorporate such factors. Therefore, in order to meet the shortcomings of the conventional models, microfluidic organ-on-a-chip systems have been developed. These systems provide a clearly defined, well-controlled physicochemical microenvironment for cell and tissue organization. Cells are exposed not only to fluid shear stresses by perfused microchannels but also forces such as mechanical cyclic strain similar to what they would normally experience in living organs during processes such as breathing as in the case of lung-on-a-chip device reported by Huh *et al.*^5^ In another example, electric fields can be incorporated into these systems to pace contractile cells.^35^ As a result, organs-on-chips demonstrate functional realism that is normally not found in other *in vitro* systems. Despite their improved functional realism, organ-on-chips devices typically require dedicated measurement setups and present specific challenges for assessing tissue barrier function. The section on “Overview of assessing barrier integrity in organs-on-chips” provides an overview of how barrier function is typically assessed in organs-on-chip systems along with their unique challenges.

Conventional TEER measurement setups (i.e., EVOM) are mostly confined to static and macroscopic cellular environments. Therefore, they are not suitable to be used in microfluidic systems due to the small scale of the devices which makes electrode placement in close proximity to the cells impossible. This leads to variations between measurements when electrodes are not firmly secured in the same positions. Integrating the electrodes directly into an organ-on-a chip model and placing the electrodes closer to the cellular monolayer can reduce the influence of electrical resistance from the cell culture medium and the noise generated by any electrode motion. Moreover, electrodes can be scaled relative to the size of the microchannel dimensions within the system; thus, compared to conventional systems, TEER can be measured with much smaller surface areas in organ-on-chip systems. However, one needs to ensure a uniform current density across a cellular monolayer.^20^

**FIG. 2.**
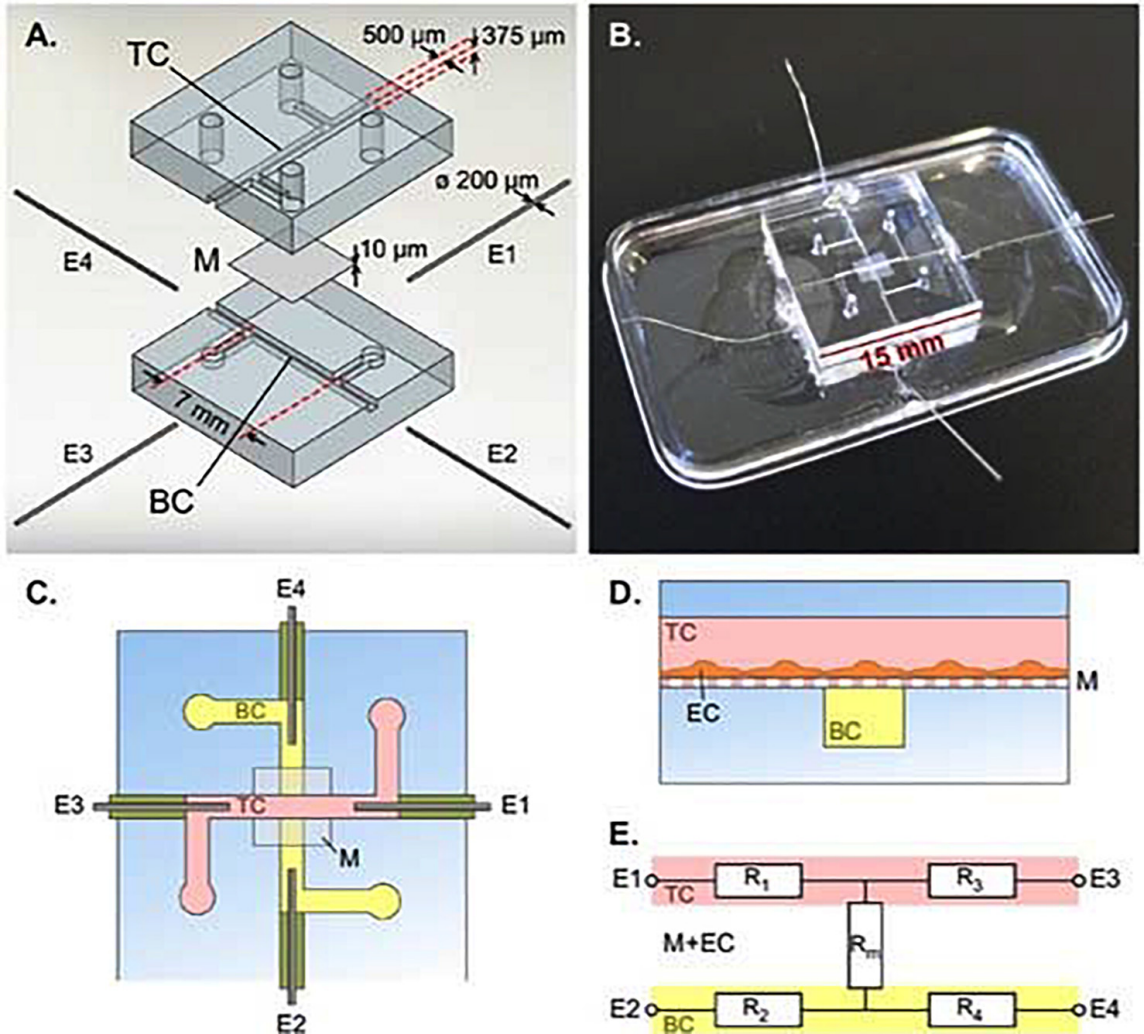
**Integrated electrodes for measuring TEER in a BBB-Chip**. (A) Exploded view of the device with top channel (TC), membrane (M), bottom channel (BC), and platinum wire electrodes (E1, E2, E3, E4). (B) Assembled device. (C) Schematic top view of the device. (D) Cross section schematic of the device showing endothelial cells (EC) cultured on membrane M in the TC. (E) Simplified equivalent circuit of the device, showing electrodes E1-E4, resistors representing the TC (R_1_ and R_3_), resistors representing the BC (R_2_ and R_4_), and resistor R_m_ representing the membrane and EC barrier. [Reproduced with permission from van der Helm *et al.*, Biosens. Bioelectron. **85**, 924 (2016). Copyright 2016 Elsevier.]

As mentioned before, organs-on-chips can incorporate physiologically relevant fluid flow to study cells in conditions that resemble the *in vivo* situation more closely. Thus, TEER has been commonly used to evaluate functionality of several barriers including BBB, gastrointestinal tract, and pulmonary tissues.^5,6,36–47^ An example of such a system is the BBB-chip reported by Van der Helm *et al.*, a multi-layered microfluidic device comprising two polydimethylsiloxane (PDMS) parts with defined microchannels, separated by a membrane made of polycarbonate, and containing 4 integrated platinum electrodes (200 *μ*m in diameter) that are inserted into the culture channels through side channels in the PDMS (Fig. 2).^44,48^ For TEER measurements, a lock-in amplifier with a probe cable circuit is coupled with two of the four electrodes. A series of six resistance values is recorded using all the possible pairs of electrodes. Subsequently, Gaussian elimination is used to determine the resistance of the cellular barrier and membrane from these six resistance values. The four-electrode system enables direct isolation of barrier resistance regardless of variations in the system (e.g*.,* temperature fluctuations and changes in medium solute concentrations) affecting the inherent resistance of the system. Resulting TEER values obtained using this method were comparable to the values obtained by conventional Transwell systems.^44^

Assessment of barrier quality of cells in organs-on-chips with TEER has been challenging due to various factors. In these systems, temperature and physical support for cell culture as well as the characteristics of the electrodes such as material, quality, and surface state have an influence on the TEER values.^40^ Non-uniform current densities are a well-established source of measurement error for TEER in any system,^40,49,50^ but are particularly important in organs-on-chips, due to the relatively low volume of medium in microfluidic channels resulting in high electrical resistance comparable to the cell layer resistance.^40,49^ In order to ensure a uniform current density and thus an equal potential drop over the entire cell culture area, one could integrate electrodes along the entire channel even though this might not be compatible with devices where mechanical deformations (e.g*.,* stretching) are applied.^40^ Alternatively, correction factors can be applied when calculating TEER from raw measurements to account for non-uniform current densities.^40,49^ Other potential sources of measurement errors are chip-to-chip variation in positioning of the electrodes and air bubbles present in microchannels (different cross-sectional areas). In addition to these physical sources of measurement errors, variations can be caused by incomplete cell coverage, even though cells of interest in the monolayer express cell-cell junction proteins. A slight gap (0.4%) in cell coverage can potentially reduce the TEER measured by 80%.^40^ It is essential to control all these sources of variation to enable comparison of TEER values between different microfluidic systems.

**FIG. 3.**
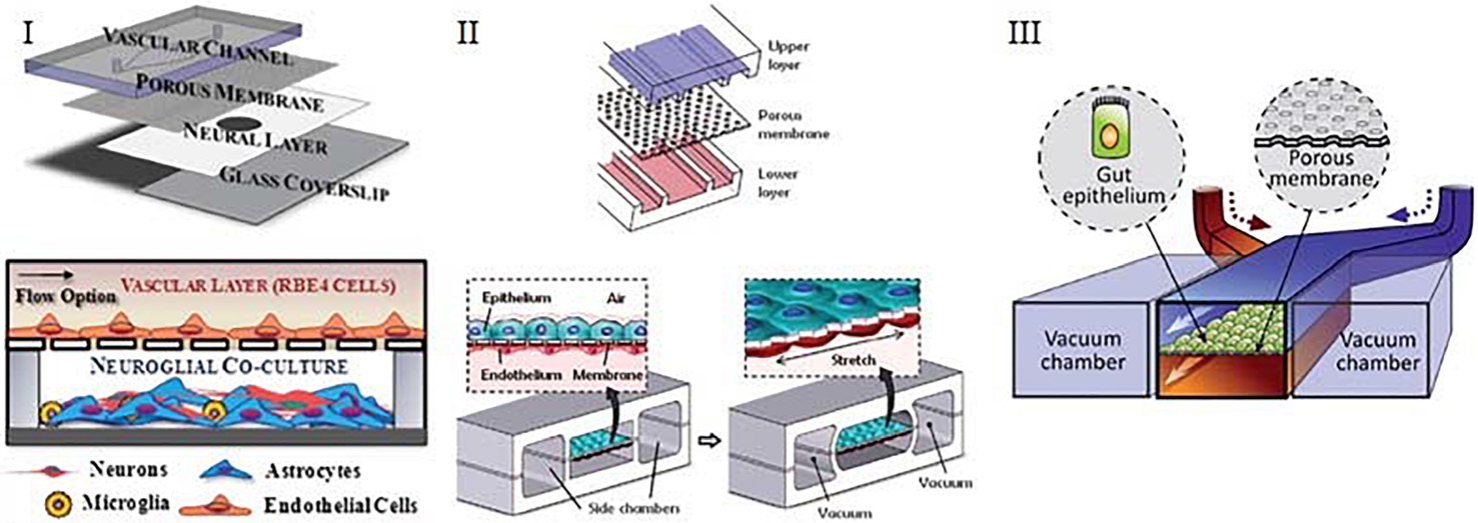
**Typical organ-on-a-chip devices containing two adjacent channels separated by a semi-permeable membrane.** (I) Device of Achyuta *et al.* that consists of 2 parts which are assembled following the cell seeding. [Reproduced with permission from Achyuta *et al.*, Lab Chip **13**, 542 (2012). Copyright 2012 The Royal Society of Chemistry.] (II) Device of Huh *et al.* that consists of two adjacent channels separated by a porous PDMS membrane. [Reproduced with permission from Huh *et al.*, Science **328**, 1662 (2010). Copyright 2010 American Association for the Advancement of Science.] (III) Design of Kim *et al.* that contains two channels, one of which seeded with gut epithelial cells, other containing the interstitial fluid. [Reproduced with permission from Kim *et al.*, Lab Chip **12**, 2165 (2012). Copyright 2012 The Royal Society of Chemistry.]

Assessment of a cellular barrier by means of resistance provides label-free, real time information, but it does require dedicated measurement setups as well as device designs. Therefore, assessment of barriers by measuring transport of tracer molecules is used more often in organ-on-chip systems. An example of such a system is the BBB-on-chip model of Achyuta *et al.*, which involves two cell types cultured on microchannels assembled into a chip (Fig. 3-I). In this device, barrier integrity was measured using diffusion of fluorescently labeled dextran (3 kDa), which was perfused in the vascular channel and collected at the neural layer. The amount of diffused dextran was measured using a plate reader.^51^ Another example of using fluorescently labeled molecules to assess barrier integrity is the lung-on-a-chip device used by Huh *et al.* (Fig. 3-II).^5^ This device is a three-layered sandwich where two adjacent channels were separated by a porous membrane. Added to the upper alveolar layer, FITC-conjugated albumin transport was measured by sampling liquid flowing through the lower channel. Similarly, Kim *et al.* demonstrated the diffusion of fluorescently labeled dextran added to the upper channel of a gut-on-chip device, by taking hourly samples from the bottom layer (Fig. 3-III).^6^ Contrary to the traditional Transwell systems, these devices are dynamic. That means culture medium is often continuously perfused through the top and bottom channels, which makes it impossible to measure an increase in concentration in the collecting channel by repeated sampling. The concentration of tracer molecules in all repeatedly collected samples will be typically be constant, and will only depend on the barrier tightness and the residence time—the time for molecules to accumulate in the fluid of the collecting compartment as it flows through the chip. The residence time is dictated by the volumetric flow rate and the volume of the collecting channel. Typically, due to continuous perfusion and the low volume of compartments, residence times are short, which means that the effective “sampling time” that can be used to estimate (dC_w_/dt)_0_ for Eq. (3) is also short. This may lead to low effective concentrations of tracers to reliably estimate the rate of increase. To overcome this issue, multiple measures can be taken: flow rates in the channels can be decreased, concentrations of tracer molecules in the source channel can be increased, and smaller tracer molecules with higher permeability coefficients can be used. Another complication of using this method in organs-on-chips is that differences in pressures or hydraulic resistances between channels, although small, might cause transport of tracer molecules through advection instead of diffusion. Therefore, one should be careful with the fluid levels in different inlets to be equal to prevent any pressure differences.

**FIG. 4.**
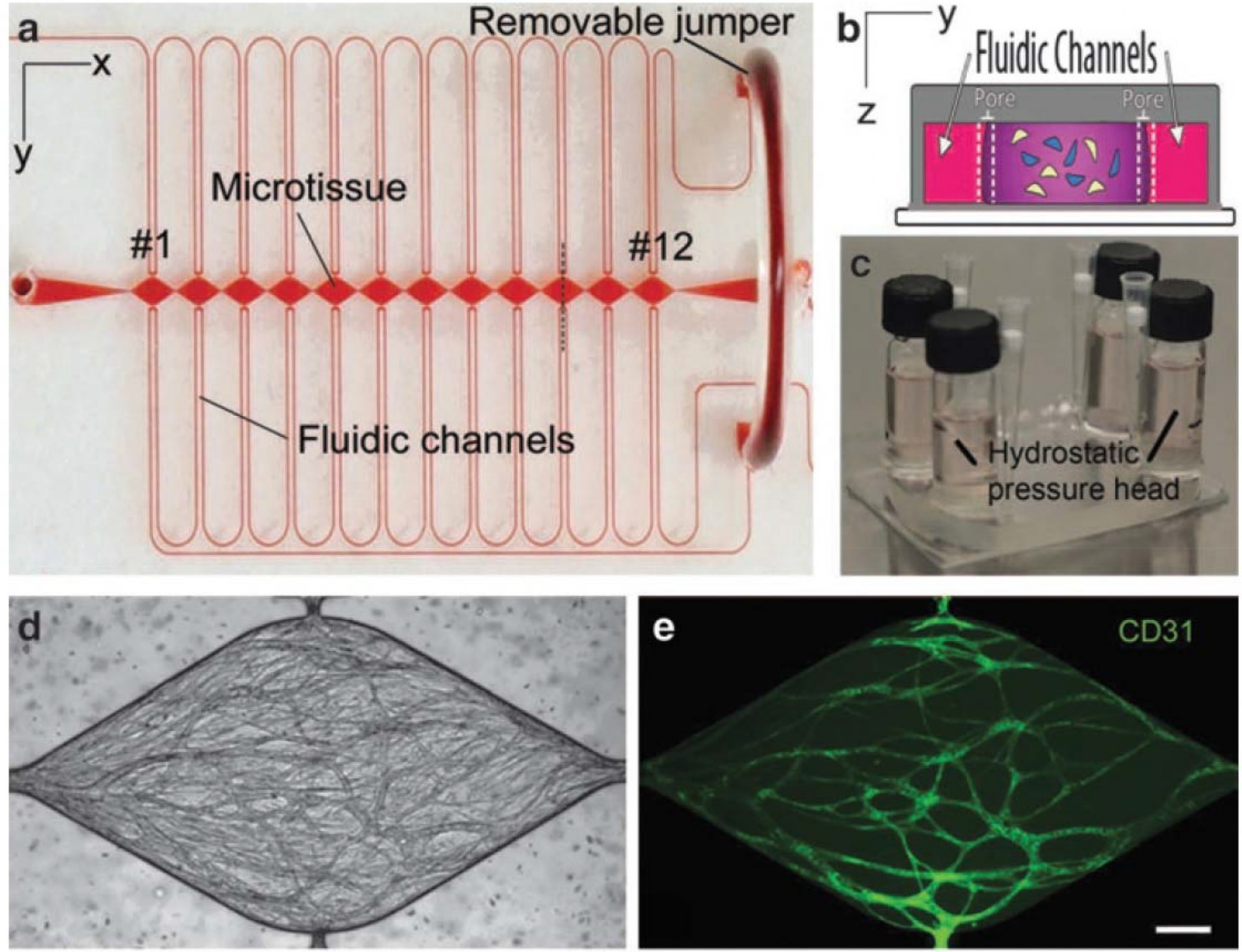
**Microfluidic system reported by Moya *et al.* for 3D vasculature modelling.** (a) PDMS based microfluidic device contains outer microfluidic channels that connect to a series of central micro-tissue chambers through a communication pore on each side. (b) Central chamber is inhabited by endothelial cells and stromal cells embedded in a fibrin matrix [cross-section of panel (a) indicated by a black dotted line]. (c) Hydrostatic pressure is necessary for media flow and is enabled by large media reservoirs. (d) and (e) Microfluidic system enables robust interconnected vessel network formation within 14–21 days (scale bar= 200 *μ*m). [Reproduced with permission from Moya *et al.*, Tissue Eng. Part C **19**, 730 (2013). Copyright 2013 Mary Ann Liebert Inc.]

Organs-on-chips are becoming progressively more three-dimensional to mimic *in vivo* tissue structure and function. This means that many organs-on-chips now contain 3D vessels or networks of vessels. Assessment of barrier function in such devices with 3D culture area geometries typically relies on imaging of fluorescent tracers because measuring TEER is currently not possible due to challenges related to integration of electrodes as well as ensuring a uniform electric field along the culture area.^52^ An example of a device with a 3D vascular architecture is provided by Moya *et al.* They reported a microfluidic device with individual cell culture chambers which were filled with endothelial cells and fibrin matrix. Cells in these chambers self-assembled into a capillary network in the presence of cell media supplemented with growth factors (Fig. 4).^53^ Permeability of vessels could be assessed by injecting fluorescent dextran (70 and 150 kDa) to the channels followed by imaging with fluorescence microscopy. Typically, this type of microscopy data is reported to make a qualitative or semi-quantitative statement about barrier function.

Still, microscopic tracking of tracer molecules can in principle be used to make quantitative statements about barrier function in organs-on-chips with 3D geometries. For instance, Herland *et al.* constructed a 3D blood vessel-on-a-chip inside with lumens created by viscous finger patterning in a collagen I matrix.^52^ Cells seeded inside these lumens created a 3D vessel. Barrier quality in these systems was evaluated by infusing a fluorescently labeled dextran (3 kDa) followed by continuous recording of fluorescent images. Using these images, the apparent permeability coefficient (P_app_) can be calculated by analyzing the total fluorescence intensity in an area and applying

$$P_{app}=\frac{1}{\Delta I}\;{\left(\frac{dI}{dt}\right)}_{0}\frac{r}{2},$$ (6)

where ΔI is the step increase in the total fluorescence intensity upon adding dextran, (dI/dt)_0_ is the initial rate of increase in intensity as dextran diffuses out of the vessels into the surrounding gel, and r is the radius of the tube.^54^ For this type of measurements, it is essential to ensure a stable monolayer of cells in the beginning of the dye addition, otherwise if diffusion of fluorescently labeled molecules is too fast to reliably establish the intensity step ΔI, and quantification will not be possible.

### Overview of assessing barrier integrity in organs-on-chips

Since their inception, a wide variety of organs-on-chip designs have been optimized to model various tissues of the human body. By the controlled incorporation of physiologically relevant forces, flows, and geometries that are also found in their native *in vivo* environment, one can better recapitulate tissue and organ level physiology. Below is an overview of different organ-on-chip systems used to assess cellular barriers of different tissues. The list includes only a small fraction of a vast number of models as the focus of this review was restricted to models investigating cellular barriers (Table I).

**TABLE I.** Overview of barrier assessment techniques in organ-on-a-chip systems.

Organ	Assessment	Co-culture	Culturing type	References
Blood-brain Barrier	TEER	No	2D	41 and 46
Blood-brain Barrier	PTT	No	2D	60 and 55
Blood-brain Barrier	PTT	Yes	2D	56 and 67
Blood-brain Barrier	PTT	Yes	3D	57 and 56
Blood-brain Barrier	PTT	No	3D	57
Blood-brain Barrier	TEER	Yes	2D	58
Blood-brain Barrier	TEER, PTT	Yes	2D	36, 46, 59, and 60
Blood-brain Barrier	TEER, PTT	Yes	3D	41, 61, and 62
Cornea	PTT	No	2D	63
Gastrointestinal Tract	PTT	Yes	2D	64 and 65
Gastrointestinal Tract	TEER	Yes	2D	66
Gut	TEER, PTT	No	2D	6
Kidney	TEER, PTT	Yes	2D	38
Liver	PTT	No	2D	67
Lung	TEER, PTT	Yes	2D	5
Lung	PTT	Yes	2D	68
Retina	PTT	Yes	2D	69
Retina	PTT	Yes	3D	70
Vasculature	PTT	No	3D	53 and 71
Multiple organs	TEER	Yes	2D	72
Multiple organs	TEER, PTT	Yes	2D	46

Following abbreviations were used to describe each of the techniques: TEER for trans-endothelial/epithelial electrical resistance and PTT for paracellular transport of tracer molecules. Each row of Table I has been classified based on the organ of interest, barrier assessment method (whether TEER, PTT, or combination), co-culture (presence of multiple cell types in the same model), and culturing type (whether cells have been cultured in hydrogels to provide a 3D microenvironment).

As it is challenging to setup TEER for 3D culture areas due to previously stated factors, it has not been preferred in many models, and instead barrier function is more often evaluated using PTT method.^56,61^ Moreover, it has been reported that the cells exposed to mechanical forces exhibit increased paracellular permeability even though TEER values for the cell layer remains stable, possibly due to increased transcytosis.^6^ On the other hand, the assessment of barrier function by TEER has unique advantages, as it can be performed continuously, non-invasively and in a controlled atmosphere. Moreover, technical proof-of-concept studies in impedance spectroscopy have demonstrated that also in 3D cell culture, electrical signals from integrated electrodes can still provide information about, e.g., cell numbers and barrier properties in chips.^73,74^ Therefore, whenever possible, a combination of both methods should be applied to ensure a reliable readout on the barriers.

### Future technical development of mimicking barriers in organs-on-chips

As is clear from Table I, many different barrier tissues have already been modeled with organ-on-chip technology. As organ-on-chip technology is developed further, the 3D culture configurations will become increasingly complex. This can already be observed in recent studies that focus on advanced 3D scaffolds for alveolus-on-chip^75^ and colon-on-chip systems.^76^ Obviously, this increasing complexity will also lead to challenges in measuring barrier function, e.g., with interpreting signals from electrical sensors. It will therefore be important to keep developing innovative read-outs and more sophisticated sensor technology, such as 3D biocompatible electrodes that can directly integrate in the cultured tissues.^77^

Most organs-on-chips are currently fabricated from PDMS, but the high gas permeability of this material makes it challenging to control specific gas pressures in an organ-on-chip system. Local control over gas concentrations is important in studying the transport of gases over barrier tissues in, for example, lung-on-a-chip or the vessel-on-a-chip systems. In addition, the barrier function of many tissues is affected by local oxygen concentrations. For example, the permeability of blood vessels changes dynamically in episodes of ischemia and reperfusion,^78^ and the barrier function of intestinal epithelium is affected by interactions with anaerobic bacteria that only survive in low-oxygen conditions.^79^ Another challenge when using PDMS-based devices is selective adsorption and absorption of molecules from the culture media, which in turn reduces their effective concentrations and ability to affect cells. This is especially important in drug efficacy or toxicology studies where compound availability to the cells is required to determine the dosage and efficiency of the drug.^80^ To reduce absorption, PDMS channel surfaces are often coated to block the passage of compounds.^81^ Alternatively, organ-on-chip systems are increasingly being manufactured with materials other than PDMS, such as polystyrene, glass, and cyclic olefin copolymer to allow control over gas pressures as well as to prevent absorption of compounds.^82,83^

Combined with the engineering of organ-on-chip systems that contain ever more physiologically relevant cues, *in vitro* barrier models will also integrate progressively more cell types. By doing so, it will be possible to mimic cellular dynamics as well as crosstalk between cell types, such as barrier tissue cells and immune cells. However, one needs to be cautious about the media compatibility when incorporating several cell types. Cells may not survive in each other's respective medium and continuous fluid flow may be needed to allow locally stable culture conditions for various cell types. Together with an increasing 3D complexity of organ-on-chip systems, this will require innovative solutions for microfluidic actuation, for example, by 3D-printing parts of organ-on-chip systems.

## CONCLUSION

Barriers exist in our bodies to maintain homeostasis and protect vital organs. Disruption of tissue barriers leads to various diseases. It is undeniable that investigation of these barriers in diseases might reveal new mechanisms and treatments. Therefore, proper *in vitro* tools are required to evaluate the integrity and characteristics of barriers. Conventional Transwell systems suffer from not fully recapitulating the complexity of the microenvironment as well as inclusion of physical forces that have an impact on the development and differentiation of cells. Microfluidic organ-on-chip systems are great tools to overcome these challenges. In addition to integrating physical stimuli, they consist of additional co-cultures with immune cells and microbes to mimic the physiological tissue environment more realistically, thus giving more accurate information about underlying organ physiology and disease mechanisms.^8^ Due to their complexity and a wide plethora of designs, it can be challenging to compare measurements performed in a specific organ-on-chip system with data from other organs-on-chips or conventional assays. To overcome this challenge, it is essential to implement assays that quantitatively measure barrier function, independent of exact system design. Assessing barrier function will be of importance especially in cases where multiple organ models are combined to create body-on-chip systems, where barrier functions influenced by other organ models (e.g*.,* via inflammation) can be studied. Incorporating such measurements in organs-on-chips may require adjustments or corrections to avoid common measurements errors, as discussed in this review.

Finally, advances in stem cell technology and access to patient-derived cells will improve the physiological relevance of current organ-on-chip models and will contribute to more realistic and patient-specific disease models. Continuous monitoring of barriers without disrupting viability of the cells in such organ-on-a-chip models will yield unique insights into mechanisms of disease, thus contributing to the development of patient-specific treatments in the context of precision medicine.
